# The diagnostic value of transbronchial lung cryobiopsy combined with rapid on-site evaluation in diffuse lung diseases: a prospective and self-controlled study

**DOI:** 10.1186/s12890-022-01898-z

**Published:** 2022-04-02

**Authors:** Xianqiu Chen, Jie Luo, Li Yang, Likun Hou, Bing Jie, Yang Hu, Jianxiong Li, Xing Jiang, Jinfu Xu, Kebin Cheng

**Affiliations:** 1 Department of Respiratory and Critical Care Medicine, Shanghai Pulmonary Hospital, School of Medicine, Tongji University, 507 Zhengmin Road, Shanghai, 200433 China; 2 Department of Oncology, Shanghai Pulmonary Hospital, School of Medicine, Tongji University, Shanghai, 200433 China; 3 Endoscopy Center, Shanghai Pulmonary Hospital, School of Medicine, Tongji University, Shanghai, 200433 China; 4 Department of Pathology, Shanghai Pulmonary Hospital, School of Medicine, Tongji University, Shanghai, 200433 China; 5 Department of Radiology, Shanghai Pulmonary Hospital, School of Medicine, Tongji University, Shanghai, 200433 China

**Keywords:** Interstitial lung disease (ILD), Transbronchial lung cryobiopsy (TBCB), Transbronchial lung biopsy (TBLB), Rapid on-site evaluation (ROSE), Complication, Diagnosis

## Abstract

**Background:**

The etiology of interstitial lung diseases (ILDs) is varied. Early diagnosis and a specific pathological type could significantly improve the prognosis. Mostly, it is difficult to make the etiology diagnosis of ILD through traditional biopsy methods. It will be of great significance to explore an effective biopsy method.

**Methods:**

The prospective study was designed to evaluate the diagnostic value of transbronchial lung cryobiopsy (TBCB) combined with rapid on-site evaluation (ROSE), compared with conventional transbronchial lung biopsy (TBLB), in a large sample of ILD patients. All patients enrolled will undergo both TBLB and TBCB procedures. The study will observe the differences in the diagnostic efficiency of pathological typing and incidence of operation-related complications between TBCB and TBLB. Besides, it will analyze the relationship between the time of biopsy and the incidence of complications, the relationship between freezing time, size of specimen, and complications. And it will evaluate the consistency of pathological, clinical, and radiology.

**Discussion:**

It may be the first time that ROSE technique will be used in the diagnosis of ILD. The results of this study will clarify the value of TBCB in the diagnosis of ILD and confirm its safety and effectiveness, which is expected to significantly improve the efficiency of diagnosis in ILD patients.

*Trail registration*: The trial was registered on the Chinese Clinical Trial Registry website (http://www.chictr.org.cn/showproj.aspx?proj=57834) (Registration number: ChiCTR2000035492).

## Background

Interstitial lung diseases (ILDs) are a group of diffuse diseases that mainly involve the pulmonary interstitial and alveolar cavity. They are characterized by progressive dyspnea, diffuse infiltrating shadows in the lungs, hypoxemia, and restrictive ventilation disorder [1, 2]. The etiology of ILD is varied, and the majority of patients with idiopathic pulmonary fibrosis (IPF) lack effective treatment, with a median survival time of 3 years [3]. If patients with ILD can be diagnosed early and have a specific pathological type, individualized treatment measures could significantly improve the prognosis. At present, the diagnosis of ILD mostly requires high resolution computerized tomography (HRCT) and bronchoalveolar lavage (BAL), lung biopsy and pathological examination by various means if necessary. The common methods of lung biopsy are transbronchial lung biopsy (TBLB), X-ray/ultrasound-guided percutaneous lung biopsy, and surgical lung biopsy (SLB). However, due to the small size of specimen by TBLB and percutaneous lung biopsy, and the extrusion and deformation of tissues, they hardly meet the needs of ILD pathological diagnosis. SLB is still the most effective biopsy method at present, but SLB may increase the risk of acute exacerbation and infection rate of ILD patients, and the mortality within 30 days is about 3–4% [4, 5]. Transbronchial lung cryobiopsy (TBCB) is a technique inserting a probe into a distal bronchial or pulmonary lesion through a bronchoscope and tearing the surrounding lung tissue to obtain the target tissue. Rapid on-site evaluation (ROSE) is a technique used when specimens are collected by puncture, biopsy, brush, etc., Related personnel are on site to conduct a rapid evaluation of the satisfaction of the obtained specimen, to make a preliminary diagnosis, priority strategy, and feedback for the next step. At present, ROSE is mainly used in the diagnosis or differential diagnosis of solid malignant tumors, tuberculosis, fungal infections, sarcoidosis, and other pulmonary diseases, but rarely applied in the diagnosis of ILD.

The prospective study was designed to evaluate the diagnostic value of TBCB combined with ROSE, compared with conventional TBLB combined with ROSE in a large sample of ILD patients. It is the first study to apply ROSE technique in the diagnosis of ILD. The results are expected to establish a new, efficient and fast diagnostic method and may promote the standardization of the pathological sampling standards and pathological diagnostic standards of ILD.

## Methods

### Study objects

According to the literatures, the estimated sensitivity rates of TBCB and TBLB methods were 80% and 60%, respectively. The estimated prevalence rate of the target subjects is 50%. Using McNemar test with PASS software to calculate the sample size, when the value of ɑ was 0.05, the sample size was 122 cases. And totally 146 hospitalized patients with diffuse lung disease were intended to be enrolled in the study with the amplification of 1.2 times. The specific criteria are as follows:

1. Inclusion criteria

(a) ILD patients with unknown reasons (more than 18 years old), have the necessity to undergo TBCB for the diagnosis; (b) Hospitalized patients need to complete arterial blood gas analysis, blood routine, blood coagulation routine, liver and kidney function, electrocardiogram, immunology examination, chest HRCT, and other examinations; (c) Good compliance and can be coordinated with this study; (d) After being fully informed of the purpose and method of the study, the patients are willing to participate in the study and sign the informed consent.

(2) Exclusion criteria

(a) Do not agree to participate in the study; (b) Have participated in other studies that could affect the observation of this study; (c) Patients who fail to complete the examination required for admission before TBCB or TBLB; (d) Chest HRCT showed non-interstitial lung disease; (e) Any conditions deemed unsuitable for inclusion by the investigators;

(3) Elimination criteria

(a) Patients who do not meet the inclusion criteria or who meet the exclusion criteria after enrollment; (b) After enrollment, patients have poor compliance and could not cooperate with the observers;

(4) Withdrawal criteria

Patients who meet the inclusion criteria and fail to complete the study for some reasons, including patients who voluntarily withdraw and are determined to withdraw by the researchers.

(5) Study end points
  - a. Primary end point: The differences in diagnostic efficiency and incidence of operation-related complications between TBCB and TBLB.
  - b. Secondary end point: Analyze the relationship between the time of biopsy and the incidence of complications; the relationship between freezing time, size of specimen and complications; The histopathological results will be handled in a multidisciplinary meeting, to analyze the consistency of pathological, clinical, and radiology.

**Fig. 1:**
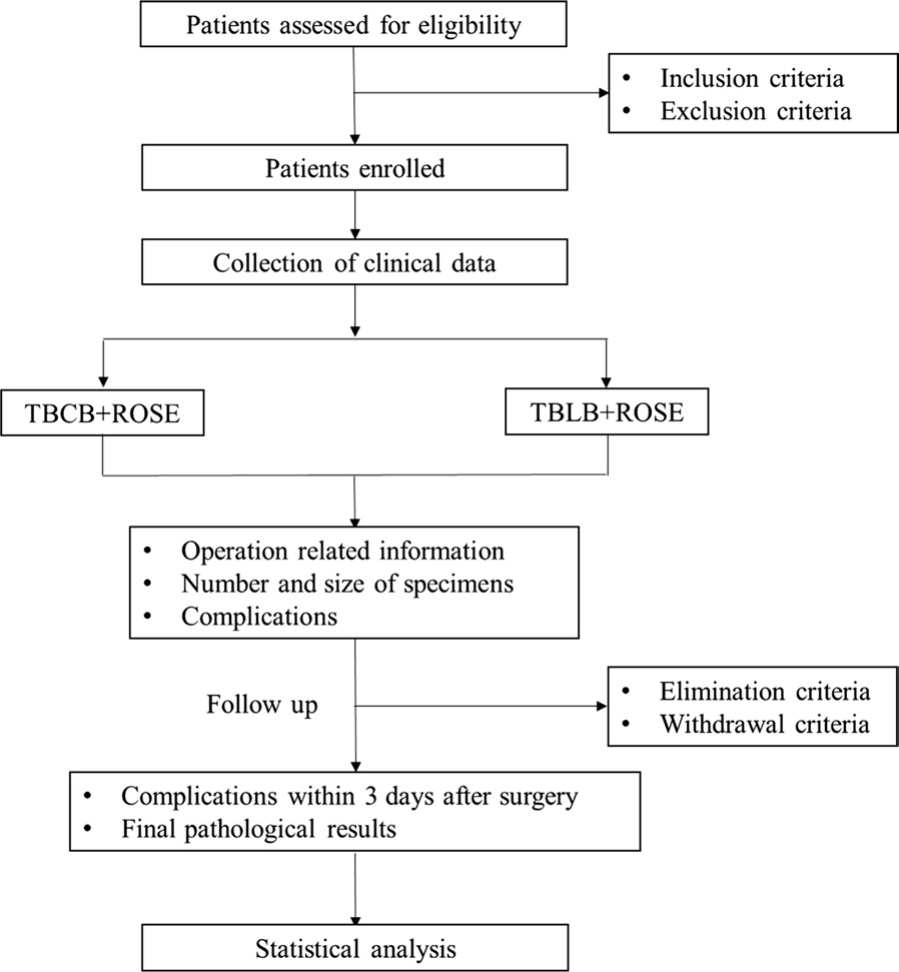
Research process. TBLB, transbronchial lung biopsy; TBCB, transbronchial lung cryobiopsy; ROSE, rapid on-site evaluation

The research process is shown in Fig. 1.

### Process of TBCB/TBLB

The patients enrolled will undergo both TBLB and TBCB procedures.

1. Tracheal intubation, mechanical ventilation, and vital signs monitoring

Sedation and analgesia. Place the bite and No. 8 tracheal catheter under the guidance of a soft bronchoscope. When the distal end of the catheter is located in the middle and lower part of the trachea, the catheter will be fixed. A monitor will monitor the vital signs.

(2) Placement of guide wire

Insert the soft bronchoscope via the nose, enter the trachea through the glottis from the trachea catheter, empty the capsule, and then enter the bronchus. Place the guide wire into the bronchus of the intended biopsy segment through the bronchoscope working passage, and exit the bronchoscope after the catheter is in its place. Fill the capsule and fix the guide wire, and then the guide wire will be strengthened by the respiratory therapist who is responsible for mechanical ventilation so that it will not displace.

(3) Placement and testing balloon

When the soft bronchoscope enters the airway through the tee and tracheal catheter, the assistant will send the balloon via the guide wire into the bronchial opening of the proposed biopsy segment or lobe. The balloon will be filled with air under direct vision and adjusted to completely close the bronchial opening of the proposed biopsy segment or lobe, and it is appropriate to remember the amount of air injected.

(4) TBCB/TBLB

**Fig. 2:**
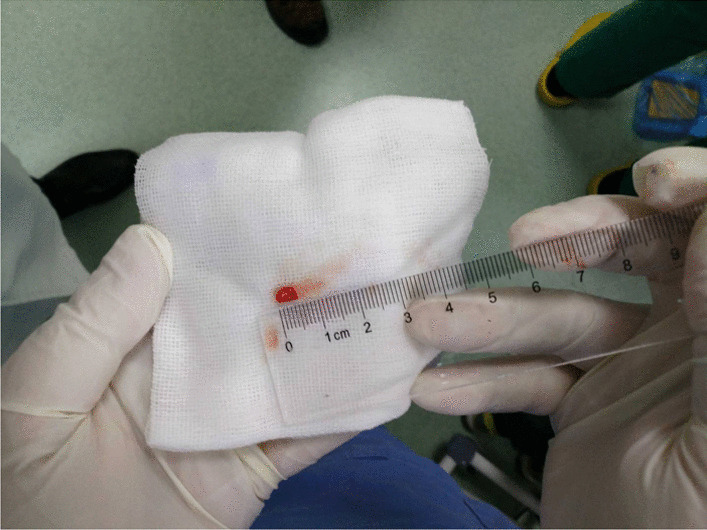
Lung tissue obtained by transbronchial lung cryobiopsy

When the endotracheal intubation is connected to the ventilator or high-frequency ventilation, bronchoscopy will be performed. First, conventional TBLB will be performed with biopsy forceps under the guidance of X-ray, and biopsies will be performed through different segmental bronchi 2 or 3 times in general. The biopsy specimens will be checked by pathologists and performed ROSE on site to guarantee the quality to continue the biopsy. After the biopsy, X-ray fluoroscopy will be performed to check whether there is pneumothorax, and the size of biopsy specimens, bleeding, pneumothorax and operation duration will be recorded. Then, TBCB will be performed at the same site. Freezing probes will be sent to the biopsy site through a bronchoscope work channel with positioning. The bronchoscope and cryogenic probe will be pulled out after refrigeration at a distance of 1 ~ 2 cm away from the pleura for 3 ~ 4 s. Take the biopsy specimen off after thawing. The bronchoscope will reenter into the bronchial to check for bleeding. Biopsy is usually done 2 ~ 3 times. Figure 2 shows the lung tissue obtained by TBCB. Biopsy specimens will be checked by pathologists and will be performed ROSE on site to guarantee the quality for further biopsy. After the biopsy, X-ray fluoroscopy will be performed to check for pneumothorax, and size of the biopsy specimen, bleeding, pneumothorax condition and operation duration will be recorded. In both operations, the next biopsy will be performed in the absence of significant bleeding and pneumothorax. To do another site of biopsy, insert a guide wire into the balloon again, and guide the balloon into the new segment by clamping the guide wire with biopsy forceps, and repeat the preceding process. After the biopsy, mechanical ventilation and tracheal intubation will be removed when the patient regains consciousness, and the patient will be sent back to the ward for monitoring and observation for 4–6 h.

***Collection of clinical data***

1. Collect relevant information about the operation, including anesthesia method, anesthesia complications, time of TBCB, time of TBLB, freezing time, and operation time;
2. Specimens’ information, including the number and size of specimens, ROSE results, and pathological results;
3. Complications caused by the operation, including bleeding, pneumothorax, infection, and acute exacerbations caused by the operation, as well as the delayed complications mentioned above during the 3 days of follow-up after the operation.
4. Collecting adverse events in the study:

iii. a. Adverse event (AE): Any adverse medical event that occurs during the observation period of a patient during the study, which does not necessarily imply a causal relationship with the procedure.
iv. b. Serious adverse event (SAE): An adverse event meeting one or more of the following criteria is considered as SAE, such as death; life threat; persistent or severe disability or permanent loss of function; hospitalization or prolonged stay due to AE; leading to congenital malformation.
v. c. Severity classification of adverse events: mild——uninduced or only causing mild discomfort, not affecting daily life and not requiring clinical management; moderate——active narration, affecting daily life, but tolerating, requiring clinical general management; severe——having objective manifestation that cannot be tolerated, surely affecting daily life, needing rest in bed and active clinical treatment.
vi. d. Relationship between adverse events and operation: it can be divided into five levels: ① definitely relevant, ② probably relevant, ③ possibly relevant, ④ probably irrelevant, and ⑤ definitely irrelevant. ①, ②, ③ are considered as operation-related adverse events.

(5) Severity of bleeding

Grade 0: no bleeding; Level 1: bleeding that can be stopped by negative pressure suction without other hemostatic measures; Level 2: bleeding that requires local injection of frozen saline, epinephrine, or balloon closure; Level 3: severe bleeding that beyond endoscopic control, resulting in hemodynamic and respiratory instability, requiring vascular intervention or surgical hemostasis, or requiring admission to intensive care unit (ICU).

### Statistical analysis

The original data will be uniformly stored in the paper version of the case report form, and the electronic data will be single-path input and checked by two researchers. The database will be input to Excel software and will be statistically analysed with SPSS 25.0 software. The measurement data of normal distribution will be expressed as X ± S, and the measurement data of skewness distribution will be expressed as M (P25, P75). The Mann-whitney U test will be used for comparison. Enumeration data will be expressed as cases and percentages, and chi-square tests will be used for comparison.

### Trial status

The study is ongoing. The protocol was approved on 27 Dec. 2021 by the Ethics Committee of Shanghai Pulmonary Hospital, Tongji University. The trial was opened for recruitment on 28 Dec. 2021; the first patient was enrolled on 28 Dec. 2021. Only one patient has been enrolled at present.

### Dissemination plans

The results will be published in high-quality peer-reviewed journals at the end of study.

## Discussion

Early diagnosis is of great significance for the prognosis of ILD patients. At present, the diagnosis of ILD mostly requires HRCT and BAL, and if necessary, lung biopsy and pathological examination by various means. SLB is still the most effective sampling method at present, but SLB may increase the risk of acute exacerbation and infection rate, and the mortality rate within 30 days was reported at 3–4% [4, 5]. The Chest Diseases Group of the Pathological Society of the Chinese Medical Association pointed out in 2018 that the pathological biopsy of ILD patients should obtain more samples and ensure a certain size. It is recommended to take samples in different lobes of one side of the lung, generally each 1 ~ 2 pieces in various degree areas of the lesions, with a depth of 1 ~ 2 cm (because of the honeycomb changes always on the edge of the lung). Each tissue is suggested to be larger than 1.0 cm×0.7 cm×0.5 cm, which could basically meet the needs of pathological diagnosis [6]. Therefore, there is an urgent need for an advanced sampling technique for ILD diagnosis that can not only meet the needs of pathological diagnosis, but also has little trauma and fewer complications.

In 2008, Hetzel et al. formally proposed the concept of TBCB and confirmed its effect and safety in the sampling of endobronchial lesions for the first time. In 2009, Babiak and Hetzel et al. further developed the technique to peripheral lung lesions, which was at first used in the diagnosis of ILD [7, 8]. Many studies on ILD have reported that the sampling size of TBCB can be as much as 3–6 times that of conventional TBLB, and the diagnostic efficacy of TBCB is significantly higher than that of TBLB (74.4 ~ 100% vs. 43.1 ~ 69.3%) [8–11]. It was found through pathological sections that target tissues obtained by TBCB had fewer pseudo-errors, could preserve more alveoli, and account for a higher proportion of nondestructive, which may be more conducive to the pathological diagnosis of ILD [12]. Compared with SLB, Hagmeyer et al. [13] conducted a comparative study for the first time in the diagnosis of ILD. TBCB was performed on 32 subjects, including 8 patients who received SLB, and TBCB was strongly consistent with the final diagnosis results of 78%. 7 of the 8 patients who received SLB had a good correlation with TBCB pathology (88%). Recent years, a number of studies have examined the diagnostic yield and safety of TBCB for ILD, with varying findings [14–19].

About the safety of TBCB, it was reported that there were no significant differences in the incidence of pneumothorax, bleeding, and bleeding severity compared with conventional TBLB [20, 21]. Yarmus et al. [22] reported for the first time that TBCB was used to evaluate 11 patients after lung transplantation under a soft microscope and achieved satisfactory results without serious complications. The CHILL study [23] reported that TBCB was associated with mild bleeding (grade 1 bleeding that requiring suction to clear) in 56.2% of patients, but only 16 cases were included in the study. These studies indicated that TBCB had good safety and efficacy in ILD, but they were all retrospective studies with small samples, and prospective studies with large samples are urgently needed in clinical practice to evaluate the diagnostic value of TBCB in ILD.

ROSE is a rapid cytological interpretation technique which accompanies sampling in real time during diagnostic interventional operations. In the sampling of the target sites, under the premise of almost no loss of tissue specimen, part of the sample is printed on the slides to make a cytological substrate, quickly stained and immediately interpreted with a special microscope integrated with clinical information. As a cell carrier, ROSE has the following functions: evaluation quality of the samples, guidance intervention means of operation in real time, forming the preliminary diagnosis or narrowing the scope of differential diagnosis, optimization the processing scheme of the target specimen, analyzing and predicting the outcomes of the disease combined with all clinical and cytological information [24]. However, ROSE has not been applied in ILD at present.

It is a prospective and self-controlled study to evaluate the diagnostic value of TBCB/TBLB combined with ROSE technology in a large sample size of ILD patients. In addition, it is the first time that ROSE technique was used in the diagnosis of ILD. The results of this study will clarify the value of TBCB in the diagnosis of ILD and confirm its safety and effectiveness, which is expected to improve the efficiency of diagnosis and improve the prognosis of ILD patients.

Abbreviations: TBLB, transbronchial lung biopsy; TBCB, transbronchial lung cryobiopsy; ROSE, rapid on-site evaluation.

## Data Availability

The datasets used and analysed during the current study will be available from the corresponding author on reasonable request.

## Abbreviations

ILD: Interstitial lung disease
IPF: Idiopathic pulmonary fibrosis
HRCT: High resolution computerized tomography
BAL: Bronchoalveolar lavage
TBLB: Transbronchial lung biopsy
SLB: Surgical lung biopsy
TBCB: Transbronchial lung cryobiopsy
ROSE: Rapid on-site evaluation
AE: Adverse event
SAE: Serious adverse event
ICU: Intensive care unit.

## References

[1] American Thoracic S, European Respiratory S, American Thoracic Society/European Respiratory Society International Multidisciplinary Consensus Classification of the Idiopathic Interstitial Pneumonias (2002). This joint statement of the American Thoracic Society (ATS), and the European Respiratory Society (ERS) was adopted by the ATS board of directors, June 2001 and by the ERS Executive Committee, June 2001. Am J Respir Crit Care Med.

[2] Bradley B, Branley HM, Egan JJ, Greaves MS, Hansell DM, Harrison NK, et al. Interstitial lung disease guideline: the British Thoracic Society in collaboration with the Thoracic Society of Australia and New Zealand and the Irish Thoracic Society (vol 63, Suppl V, pg v1, 2008). Thorax. 2008; 63:1029. 10.1136/thx.2008.101691 18757459

[3] Ley B, Collard HR, King TE (2011). Clinical course and prediction of survival in idiopathic pulmonary fibrosis. Am J Respir Crit Care Med.

[4] Raghu G, Collard HR, Egan JJ, Martinez FJ, Behr J, Brown KK (2011). An official ATS/ERSARS/ALAT statement: idiopathic pulmonary fibrosis: evidence-based guidelines for diagnosis and management. Am J Respir Crit Care Med.

[5] Kaarteenaho R (2013). The current position of surgical lung biopsy in the diagnosis of idiopathic pulmonary fibrosis. Respir Res.

[6] Association P, Chinese Medical Association, Thoracic Diseases Group (2018). Chinese specification for clinical-radiologic-pathological diagnosis of idiopathic pulmonary fibrosis. Chin J Pathol.

[7] Hetzel J, Hetzel M, Hasel C, Moeller P, Babiak A (2008). Old meets modern: the use of traditional cryoprobes in the age of molecular biology. Respiration.

[8] Babiak A, Hetzel J, Krishna G, Fritz P, Moeller P, Balli T (2009). Transbronchial cryobiopsy: a new tool for lung biopsies. Respiration.

[9] Griff S, Ammenwerth W, Schonfeld N, Bauer TT, Mairinger T, Blum TG (2011). Morphometrical analysis of transbronchial cryobiopsies. Diagn Pathol.

[10] Chou CL, Wang CW, Lin SM, Fang YF, Yu CT, Chen HC (2013). Role of flexible bronchoscopic cryotechnology in diagnosing endobronchial masses. Ann Thorac Surg.

[11] Pajares V, Puzo C, Castillo D, Lerma E, Montero MA, Ramos-Barbon D (2014). Diagnostic yield of transbronchial cryobiopsy in interstitial lung disease: a randomized trial. Respirology.

[12] Schumann C, Hetzel J, Babiak AJ, Merk T, Wibmer T, Moller P (2010). Cryoprobe biopsy increases the diagnostic yield in endobmnchial tumor lesions. J Thorac Cardiovasc Surg.

[13] Hagmeyer L, Theegarten D, Wohlschlager J, Treml M, Matthes S, Priegnitz C (2016). The role of transbronchial cryobiopsy and surgical lung biopsy in the diagnostic algorithm of interstitial lung disease. Clin Respir J.

[14] Tomassetti S, Wells AU, Costabel U, Cavazza A, Colby TV, Rossi G (2016). Bronchoscopic lung cryobiopsy increases diagnostic confidence in the multidisciplinary diagnosis of idiopathic pulmonary fibrosis. Am J Respir Crit Care Med.

[15] Ussavarungsi K, Kern RM, Roden AC, Ryu JH, Edell ES (2017). Transbronchial cryobiopsy in diffuse parenchymal lung disease: retrospective analysis of 74 cases. Chest.

[16] Wälscher J, Groß B, Eberhardt R, Heussel CP, Eichinger M, Warth A (2018). Transbronchial cryobiopsies for diagnosing interstitial lung disease: real-life experience from a tertiary referral center for interstitial lung disease. Respiration.

[17] Ravaglia C, Wells AU, Tomassetti S, Gurioli C, Gurioli C, Dubini A (2019). Diagnostic yield and risk/benefit analysis of trans-bronchial lung cryobiopsy in diffuse parenchymal lung diseases: a large cohort of 699 patients. BMC Pulm Med.

[18] Troy LK, Grainge C, Corte TJ, Williamson JP, Vallely MP, Cooper WA (2020). Diagnostic accuracy of transbronchial lung cryobiopsy for interstitial lung disease diagnosis (COLDICE): a prospective, comparative study. Lancet Respir Med.

[19] Cooper WA, Mahar A, Myers JL, Grainge C, Corte TJ, Williamson JP (2021). Cryobiopsy for identification of usual interstitial pneumonia and other interstitial lung disease features. Further Lessons from COLDICE, a Prospective Multicenter Clinical Trial. Am J Respir Crit Care Med.

[20] Ganganah O, Guo SL, Chiniah M, Li YS (2016). Efficacy and safety of cryobiopsy versus forceps biopsy for interstitial lung diseases and lung tumours: A systematic review and meta-analysis. Respirology.

[21] Sharp C, McCabe M, Adamali H, Medford AR (2017). Use of transbronchial cryobiopsy in the diagnosis of interstitial lung disease-a systematic review and cost analysis. QJM.

[22] Yarmus L, Akulian J, Gilbert C, Illei P, Shah P, Merlo C (2013). Cryoprobe transbronchial lung biopsy in patients after lung transplantation: a pilot safety study. Chest.

[23] Wahihi MM, Argento AC, Mahmood K, Shofer SL, Giovacchini C, Pulsipher A (2021). Comparison of Forceps, Cryoprobe, and Thoracoscopic Lung Biopsy for the Diagnosis of Interstitial Lung Disease - The CHILL Study. Respiration.

[24] National Health and Family Planning Commission, Cross-Straits Medical and Health Exchange Association (2017). Respiratory Professional Committee, Chinese Medical Association, Tuberculosis Association, Respiratory Endoscopy Professional Committee, Chinese Medical Doctor Association, Pediatrics Branch, Endoscopy Professional Committee, Chinese Society of Bronchial and Interventional Lung Diseases, Tianjin Medical Doctors Association, Sleep Medicine Professional Committee, Respiratory and critical Care Group. Clinical practice guidelines for rapid field evaluation of diagnostic interventional pulmonary disease. Tianjin Med J.

