# Correction to: The diagnostic value of transbronchial lung cryobiopsy combined with rapid on-site evaluation in diffuse lung diseases: a prospective and self-controlled study

**DOI:** 10.1186/s12890-022-01946-8

**Published:** 2022-04-20

**Authors:** Xianqiu Chen, Jie Luo, Li Yang, Likun Hou, Bing Jie, Yang Hu, Jianxiong Li, Xing Jiang, Jinfu Xu, Kebin Cheng

**Affiliations:** 1 Department of Respiratory and Critical Care Medicine, Shanghai Pulmonary Hospital, School of Medicine, Tongji University, 507 Zhengmin Road, Shanghai, 200433 China; 2 Department of Oncology, Shanghai Pulmonary Hospital, School of Medicine, Tongji University, Shanghai, 200433 China; 3 Endoscopy Center, Shanghai Pulmonary Hospital, School of Medicine, Tongji University, Shanghai, 200433 China; 4 Department of Pathology, Shanghai Pulmonary Hospital, School of Medicine, Tongji University, Shanghai, 200433 China; 5 Department of Radiology, Shanghai Pulmonary Hospital, School of Medicine, Tongji University, Shanghai, 200433 China

## Correction to: BMC Pulmonary Medicine (2022) 22:124 10.1186/s12890-022-01898-z

Following publication of the original article [1], the authors flagged that the authors Xianqiu Chen and Jie Luo had erroneously not been marked as equal contributors in the article.

The published article has been updated and the corrected author list can be seen in this correction.
